# Special Issue on “NMR-Based Metabolomics and Its Applications Volume 2”

**DOI:** 10.3390/metabo10020045

**Published:** 2020-01-26

**Authors:** Flaminia Cesare Marincola, Luisa Mannina

**Affiliations:** 1 Dipartimento di Scienze Chimiche e Geologiche, Cittadella Universitaria di Monserrato, Università di Cagliari, SS 554, 09042 Monserrato, Italy; 2 Dipartimento di Chimica e Tecnologie del Farmaco, Sapienza Università di Roma, P.le Aldo Moro 5, 00185 Rome, Italy

**Keywords:** metabolomics, NMR

## Abstract

Over the last decade, the number of scientific publications in the metabolomics area has increased exponentially. The literature includes ~29,000 contributions (articles and reviews) during the period of 2009–2019, revealing metabolomics applications in a wide range of fields, including medical, plant, animal, and food sciences (this bibliographic data were retrieved from the SCOPUS database, searching “metabolomics” in keywords). The high applicability of this approach is due to its ability to qualitatively and quantitatively characterize the chemical profile of all the low molecular weight metabolites (metabolome) present in cells, tissues, organs, and biological fluids as end products of the cellular regulatory pathways. Thus, providing a snapshot of the phenotype of a biological system, metabolomics offers useful contributions to a comprehensive insight into the functional status of human, animal, plant, and microbe organisms. The contributions collected in this Special Issue (12 articles, one review and one technical report) report on the recent technical advances and practical applications of NMR spectroscopy to metabolomics analyses.

The concept of metabonomics as an analytical approach to simultaneously profile metabolite levels in biofluids for applications in clinical and metabolic biochemistry may be dated to the end of 1980s when Nicholson and Wilson, in a review on “…the application of magnetic resonance methods to investigate the biochemical composition of body fluids that are secreted and excreted by man and animal…”, provided an farsighted overview of the potential use of this spectroscopic technique to study issues of biomedical relevance [1]. Only later, the “metabonomics” term was officially introduced by Nicholson et al. in 1999 [2] and is now used interchangeably with “metabolomics”, a term defined by Fiehn in 2002 [3]. Since then, thanks to the rapid development of analytical techniques and data analysis methods, metabolomics investigations have increased exponentially with relevant applications to the study of cell metabolism, not only in human biology but also in other research fields such as environmental sciences, marine biology, microbiology, and food science.

The theme of this Special Issue has been chosen with the aim of providing an overview of NMR metabolomics’ usefulness in different research areas. It includes 14 contributions (12 articles, one review and one technical report). We refer the interested reader to specialized reviews for a detailed discussion of NMR and pattern recognition methods in metabolomics [4].

The first contribution of this issue arises from Girelli et al. [5], who investigated the possibility of assessing Tuscan PGI (Protected Geographical Indication) monocultivar extra virgin olive oil (EVOO) classification by using ^1^H-NMR metabolic profile databases and multivariate statistical analysis (MVA) in addition to farmer declarations. A total of 202 micromilled oil samples obtained from genetically certified localized trees were analyzed. The findings pointed out a high variability of EVOO metabolome depending on the PGI allowed local cultivars and the high heterogeneity of the pedoclimatic conditions characteristic of the region.

Another example of NMR metabolomics application to EVOO was provided by Ingallina et al. [6]. In total, 303 samples of EVOO from nine Italian regions over three consecutive harvesting years were analyzed by ^1^H-NMR to investigate the presence of biomarkers of EVOOs origin (geographical area and variety), insensitive to seasonal and/or climatic changes. The linear discriminant analysis (LDA) of NMR data provided a very good classification model of oils in terms of the three geographical macroareas under investigation: Northern, Central-Southern and Island regions. Additionally, a hierarchical approach, based on breaking the overall classification problem down into a series of smaller submodels, was tested in order to differentiate the regions within each of the three identified macroareas.

The food metabolome was the object of two other contributions. A combined use of traditional (ion chromatography, dynamic headspace, sensory evaluation) and cutting-edge NMR technology was used by Iaccarino et al. for a comprehensive metabolite and sensory profiling of apple juices from 86 apple cultivars [7]. Correlations and differences between the data of different nature as well as clusters of cultivars having similar chemical and/or unique sensory properties were explored by multivariate data analysis for identifying cultivars for the production of “vintage juices”. In another article, Lemaire-Chamley et al. [8] provided hypotheses about tissue-specific metabolic regulations of tomato, a model for fleshy fruits. The proportions and compositions of all tissues of samples of the same tomato’s fruits were characterized during fruit development by using complementary analytical strategies, including ^1^H-NMR profiling. This approach showed that the largely studied pericarp tissue represents about half of the entire fruit only and the composition of each fruit tissue changed during fruit development with common and specific trends. Furthermore, it revealed compositional proximities within and between tissues.

The potential value of NMR metabolomics as an analytical tool in toxicological and physiological studies of endangered wildlife was the topic of the article from Bembenek-Bailey et al. [9]. The authors investigated the impact of crude oil and/or Corexit exposure on the metabolic profile of hatchling loggerhead sea turtle (*Caretta caretta*). The aqueous and lipophilic extracts of skeletal muscle, heart, and liver tissues from experimentally exposed animals were compared with that of seawater control, evidencing in particular the impact of oils on skeletal muscle and liver metabolisms.

Staying on animal biology, Zhu et al. [10] characterized, for the first time, the metabolic profiles of yak (*Bos grunniens*) serum, feces, and urine by using ^1^H-NMR to serve as a reference guide for the healthy yak milieu.

Two research articles in this Special Issue focused on topics of interest for neonatal sciences. One is that from Dessì et al. [11], who explored the potential use of ^1^H-NMR to characterize the colostrum of 58 mothers that delivered neonates at terms that were appropriate, small, or large for gestational age. The data analysis evidenced a clear natural separation of samples in two groups based on their oligosaccharide composition and thus the mother phenotype: secretory and nonsecretory. The other contribution was provided by Alinaghi et al. [12]. This study was thought to test the hypothesis that neonatal sepsis induces systemic metabolic alterations that rapidly affect metabolic signatures in immature brain and cerebrospinal fluid (CSF), and that early colostrum feeding may modulate the metabolome. Then, plasma, CSF, and brain tissue samples were collected after 24 h from cesarean-delivered preterm pigs with uncontrolled bloodstream infection. Nine infected piglets received total parenteral nutrition (*n* = 9), while ten were fed with enteral supplementation with bovine colostrum. The metabolic profiles of samples were compared with those of seven uninfected pigs receiving parenteral nutrition (i.e., controls). The results revealed associations between infection and metabolic changes related to the glycolysis and tricarboxylic acid cycle. Furthermore, attenuation of hypoxia-related changes in systemic and cerebral energy metabolism in the presence of oral colostrum supplementation suggested a protective role in the regulation of inflammatory responses.

The usefulness of NMR cell culture metabolomics to define characteristic metabolic phenotypes was shown by two groups. Fuchs et al. [13] used metabolomics to investigate the metabolic modulation of human macrophages (MΦs) following activation with proinflammatory or anti-inflammatory stimuli relative to resting MΦs (rif). The results from this study highlighted significant perturbation of glycolysis, lactate fermentation, the TCA cycle, oxidative stress, and de novo glycerophospholipid synthesis within the Kennedy pathway. Primasová et al. [14] employed HR-MAS to explore the mode of action of diruthenium trithiolato complex [(*p*-MeC_6_H_4_*^i^*Pr)_2_Ru_2_(SC_6_H_4_-*p*-Bu*^t^*)_3_]^+^ (DiRu-1), a metal-based drug with significant cytotoxicity against different cancer cell lines, on ovarian cancer cell line A2780 and on its cis-Pt resistant variant A2780cisR.

An upgrade of the progresses of magic-angle spinning (MAS) technique in the analysis of microscopic specimens (µMAS) and its potential use in metabolomics were the topics of the minireview of Luca-Torres and Wong [12].

Sobolev et al. [15] investigated the effect of the consumption of high fat/high glycemic load (HF-HGL) meals, including blueberries, on the inflammatory state of overweight/obese patients with metabolic syndrome by using a ^1^H-NMR-based metabolomics approach together with assays of inflammatory stress (real-time PCR). The effects of blueberry addition to a HF-HGL meal were monitored at two and four hours after the meals. The analysis of urine metabolome highlighted a positive impact of blueberries supplementation on the postprandial inflammation response, revealing the temporal kinetics of pro- and anti-inflammatory signaling events that may be important therapeutic targets for inflammatory diseases.

The present issue includes also technical papers. Zhu et al. [16] proposed a ^1^H-NMR signal recognition procedure to guide signal assignment by means of point-by-point univariate analysis. The authors showed how the resulting p-values can lead to a spectrum-like representation of surprising effectiveness in guiding the operator visual inspection. The application note of Tabatabaei-Anaraki et al. [17] offers a simple solution to convert 2D ^1^H-^13^C heteronuclear single quantum correlation (HSQC) data into a 1D “spikelet” format that can be read by software packages that can only handle 1D NMR data, preserving the 2D spectral information and dispersion.

We would like to thank all people who have contributed to this Special Issue: the authors for sharing the results of their investigations; the reviewers for improving the quality of the submitted manuscripts; the *Metabolites* editorial office for the fundamental assistance. We hope that readers will benefit from reading this Special Issue.
